# Association study of polymorphisms in the excitatory amino acid transporter 2 gene (*SLC1A2*) with schizophrenia

**DOI:** 10.1186/1471-244X-4-21

**Published:** 2004-08-06

**Authors:** Xiangdong Deng, Hiroki Shibata, Hideaki Ninomiya, Nobutada Tashiro, Nakao Iwata, Norio Ozaki, Yasuyuki Fukumaki

**Affiliations:** 1 Division of Disease Genes, Research Center for Genetic Information, Medical Institute of Bioregulation, Kyushu University, Fukuoka 812-8582, Japan; 2 Fukuoka Prefectural Dazaifu Hospital Psychiatric Center, Dazaifu, Fukuoka, Japan; 3 Department of Neuropsychiatry, Graduate School of Medical Sciences, Kyushu University, Fukuoka 812-8582, Japan; 4 Department of Psychiatry, Fujita Health University School of Medicine, Toyoake, Aichi 470-1192, Japan; 5 Department of Psychiatry, Graduate School of Medicine, Nagoya University, Nagoya, Japan

## Abstract

**Background:**

The glutamatergic dysfunction hypothesis of schizophrenia suggests that genes involved in glutametergic transmission are candidates for schizophrenic susceptibility genes. We have been performing systematic association studies of schizophrenia with the glutamate receptor and transporter genes. In this study we report an association study of the excitatory amino acid transporter 2 gene, *SLC1A2 *with schizophrenia.

**Methods:**

We genotyped 100 Japanese schizophrenics and 100 controls recruited from the Kyushu area for 11 single nucleotide polymorphism (SNP) markers distributed in the *SLC1A2 *region using the direct sequencing and pyrosequencing methods, and examined allele, genotype and haplotype association with schizophrenia.The positive finding observed in the Kyushu samples was re-examined using 100 Japanese schizophrenics and 100 controls recruited from the Aichi area.

**Results:**

We found significant differences in genotype and allele frequencies of SNP2 between cases and controls (*P *= 0.013 and 0.008, respectively). After Bonferroni corrections, the two significant differences disappeared. We tested haplotype associations for all possible combinations of SNP pairs. SNP2 showed significant haplotype associations with the disease (*P *= 9.4 × 10^-5^, *P *= 0.0052 with Bonferroni correction, at the lowest) in 8 combinations. Moreover, the significant haplotype association of SNP2-SNP7 was replicated in the cumulative analysis of our two sample sets.

**Conclusion:**

We concluded that at least one susceptibility locus for schizophrenia is probably located within or nearby *SLC1A2 *in the Japanese population.

## Background

Schizophrenia is a severe mental disorder characterized by hallucinations, delusions, disorganized thoughts, and various cognitive impairments. The life-time prevalence is about 1%, and genetic factors were known to play a critical role in its pathogenesis [1]. Based on the fact that phencyclidine (PCP) induces schizophreniform psychosis, a glutamatergic dysfunction hypothesis has been proposed for the pathogenesis of schizophrenia [2-4]. This hypothesis has been supported by recent multiple reports of association of schizophrenia with glutamate receptor genes and with the genes related to glutamatergic transmission, such as *G72 *and *NRG1 *[5-10].

In addition, other synaptic elements related to glutamate, such as excitatory amino acid transporters (EAATs), also potentially affect glutamatergic neurotransmission. EAATs maintain extracellular glutamate concentrations within physiological levels by reuptaking the synaptically released glutamate. A deficient uptake has been implicated in the pathogenesis of ischemic brain damage [11] and may be involved in neurodegenerative diseases such as amyotrophic lateral sclerosis (ALS) [12]. Recently significant increases of mRNA expression of EAAT1 and EAAT2 have been reported in the thalamus of schizophrenics, suggesting the possibility that an excessive glutamate uptake is involved in schizophrenia [13]. On the other hand, a significant decrease of EAAT2 mRNA expression was observed in the parahippocampal gyrus of schizophrenics [14]. Therefore the EAAT genes are reasonable candidates for schizophrenia, as well as glutamate receptor genes.

The EAATs family consists of five members (EAAT1-EAAT5). Their cellular localizations are different: EAAT1 and EAAT2 are astroglial, whereas EAAT3 EAAT4 and EAAT5 are neuronal [25]. Since EAAT2 accounts for approximately 90% of glutamate reuptake in the rodent forebrain [16,17], we focused on the EAAT2 gene (*SLC1A2*) in association studies of schizophrenia. *SLC1A2 *has been mapped to 11p13-p12 [18] and consists of 11 exons spanning over 165 kb. In this study we tested associations of schizophrenia with 11 SNPs distributed in *SLC1A2 *with an average interval of 15.9 kb. To enhance the detection power of the study, we also examined the haplotype associations of the SNPs with the disease.

## Methods

### Human subjects

Blood samples were obtained from unrelated Japanese individuals who had provided written informed consent. We used two Japanese sample sets in this study. In the first one, Kyushu samples, 100 schizophrenia patients (mean age 49.5; 44.0% female) were recruited from hospital in the Fukuoka and Oita areas and 100 healthy unrelated controls (mean age 51.2; 44.0% female) were recruited from the Fukuoka area. In the initial SNP selection process, we used another 16 Japanese samples which are recruited in the Fukuoka area and informed in the same way. In the second one, Aichi samples, 100 schizophrenia patients (mean age 34.4; 44% female) and 100 healthy unrelated controls (mean age 39.9; 45% female) were collected in the Aichi area about 600 km east of Fukuoka. All patients fulfilled the DSM-IV criteria for schizophrenia [19]. All of the case and control samples are ethnically Japanese. DNA samples were purified from whole peripheral blood by the method previously described [20]. This study was approved by the Ethics Committee of Kyushu University, Faculty of Medicine and the Fujita Health University Ethics Committee.

### SNP selection in the SLC1A2 region

**Figure 1:**
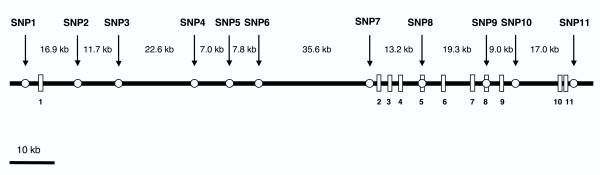
**Genomic organization of *SLC1A2 *and locations of the SNPs. **Exons are shown as vertical bars with exon numbers. Eleven SNPs are indicated by circles. Distances between the SNPs are indicated above with kb.

We retrieved the primary SNP information from the dbSNP database . Assuming the same size of the half length of linkage disequilibrium (LD) (60 kb) as reported in Caucasians [21], we initially intended to select common SNPs every 50 kb in *SLC1A2*. We tested 22 candidate SNPs including all of the exonic SNPs, in the 16 healthy Japanese samples by the direct sequencing method. Out of the 22 SNPs we selected the following 7 common SNPs with minor allele frequencies over 10% for further analyses: SNP1, rs1923295; SNP3, rs4534557; SNP6, rs1885343; SNP8, rs752949; SNP9, rs1042113; SNP10, rs3838796; SNP11, rs1570216. We also identified a novel SNP, SNP7, in intron 1 (conting location: 34105026). After the LD analyses described below, we noticed LD gaps (*D*' < 0.3) of the initial SNP set and examined additional 20 candidate SNPs. Out of the 20 SNPs, we selected the following 3 SNPs to fill the LD gaps: SNP2, rs4755404; SNP4, rs4756224; SNP5, rs1923298. The locations of the total 11 SNPs are shown in Figure 1.

### Genotyping

Eleven SNPs were amplified as 11 individual fragments by PCR using the primers shown in Table 1 - additional file 1. The reaction mixture for PCR was prepared in a total volume 10 μl with 5 ng of genomic DNA, 10 pmol of each primer (4 pmol of SNP3), 2.5 mM of MgCl_2_, 0.2 mM of each dNTP and 0.25 U of *Taq *DNA polymerase. An initial denaturing step of 1 min at 94°C was followed by 30, 35 or 40 cycles of 94°C for 30 sec, appropriate annealing temperature for 30 sec and 72°C for 30 sec. A final extension step was carried out at 72°C for 7 min. The nucleotide sequences of each primer, PCR conditions and genotyping methods for each SNP are shown in Table 1 - additional file 1. We genotyped SNP3 by pyrosequencing analysis on a PSQ™96MA Pyrosequencer according to the manufacturer's specifications with a biotinylated reverse primer (5'-CGCCTACTCCTGGTGACTTC-3'), and the sequencing primer (5'-CGCCCCCATGTGT-3'). The other 10 SNPs were genotyped by direct sequencing, as previously described [7]. The raw data of direct sequencing were compiled on PolyPhred [22].

### Statistical analyses

To control genotyping errors, Hardy-Weinberg equilibrium (HWE) was checked in the control samples by the χ^2^-test (d.f. = 1). We evaluated the statistical differences in genotype and allele frequencies between cases and controls by the χ^2^-test (d.f. = 2) and the Fisher's exact probability test (d.f. = 1), respectively. The magnitude of LD was evaluated in *D*' and *r*^2 ^using the haplotype frequencies estimated by the EH program, version 1.14 [23]. Statistical analysis of the haplotype association was carried out as previously described [24]. The significance level for all statistical tests was 0.05.

## Results

### Genotyping and SNP association analysis

We selected 11 SNPs at average interval of 15.9 kb to cover the entire *SLC1A2 *region with LD as described in Materials and Methods. Table 2 - additional file 2.  shows the results of genotype and allele frequencies of SNPs in case and control samples. No significant deviation from HWE in control samples was observed (data not shown). SNP2 showed significant differences in genotype (*P *= 0.013) and allele (*P *= 0.008) frequencies between cases and controls. After Bonferroni corrections, these two *P *values became non-significance levels (*P*_corr _= 0.143, *P*_corr _= 0.088, respectively).

### Pairwise linkage disequilibrium and haplotype association analyses

We compared the magnitude of LD for all possible pairs of the 11 SNPs in controls by calculating *D*' and *r*^2 ^(Table 3 - additional file 3. , upper diagonal), because LD around common alleles can be measured with a modest sample size of 40–50 individuals to a precision equal to 10–20% of the asymptotic limit [19]. We observed relatively strong LD (*D*' > 0.8) in the seven combinations: SNP4-SNP5 (*D*' = 0.800), SNP7-SNP8 (*D*' = 0.877), SNP8-SNP9 (*D*' = 0.925), SNP4-SNP11 (*D*' = 0.838), SNP5-SNP11(*D*' = 0.999), SNP7-SNP11 (*D*' = 0.816), SNP9-SNP11 (*D*' = 0.819). Modest LD (*D*' > 0.4) was observed in the combinations of adjacent SNPs except for SNP5-SNP6 (*D*' = 0.286) in the control samples. However, modest LD was detected in cases in the SNP5-SNP6 combination (*D*' = 0.497).

We constructed pairwise haplotypes for all of the 55 possible SNP pairs (Table 3 - additional file 3. , lower diagonal). We observed significant associations with schizophrenia in eight combinations: SNP2-SNP3 (*P *= 0.0021), SNP2-SNP4 (*P *= 0.0274), SNP2-SNP5 (*P *= 0.0054), SNP2-SNP6 (*P *= 0.0178), SNP2-SNP7 (*P *= 9.4 × 10^-5^), SNP2-SNP9 (*P *= 0.0354), SNP2-SNP10 (*P *= 0.0089) and SNP2-SNP11 (*P *= 0.0216). The combination of SNP2-SNP7 was the only one remained significant after Bonferroni correction (*P*_corr _= 0.0052).

### Cumulative analysis using the second sample set

In this study, we detected significant associations of one haplotype in the *SLC1A2 *region with schizophrenia in the Kyushu samples. To confirm the positive finding, we investigated the second Japanese sample set recruited from the Aichi area. Although significant association of the disease was observed with neither genotype, allele frequencies of SNP2 (*P *= 0.195, *P *= 0.178, respectively), nor haplotypes of SNP2-SNP7 (*P *= 0.084) in the second sample set, the significant haplotype association of SNP2-SNP7 was replicated in the cumulative analysis including the two sample sets (*P *= 5.0 × 10^-4^) (Table 4 - additional file 4. ).

## Discussion

*SLC1A2 *is located on the chromosomal region of 11p13-p12, to which no evidence has been reported for linkage of schizophrenia, [25,26]. However, there is still a possibility that *SLC1A2 *is a candidate for schizophrenia susceptibility genes, because linkage studies could only detect genes with the large genotype relative risk [27]. We carried out the genotyping of 100 cases and 100 controls for 11 SNPs, which were selected to cover the entire *SLC1A2 *region with LD. Since minor allele frequencies of each SNP we tested ranges from 0.220 to 0.485, the expected detection power of our case-control study is from 0.89 to 0.94 for the susceptibility gene assuming 2 for genotype relative risk [28].

**Figure 2:**
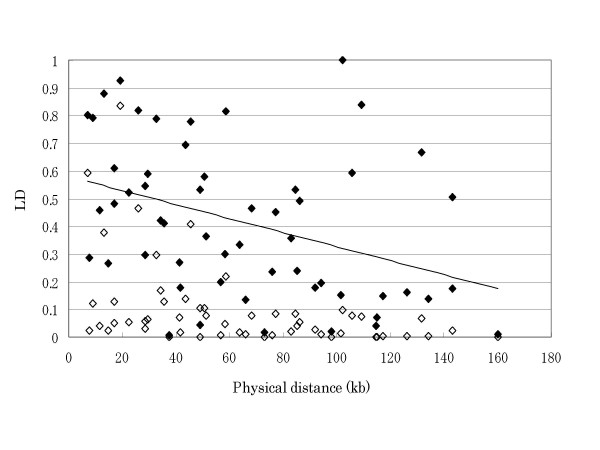
**A plot of pairwise linkage disequilibrium (LD) vs. physical distance between the SNPs in the *SLC1A2 *region. ***D*' were plotted with filled diamonds, and *r*^2 ^with open diamonds. From the regression line, the half-length of LD was estimated to be 31.8 kb in the *SLC1A2 *region.

Modest LD (*D*' = 0.925 ~ 0.409) was observed in the combinations of neighboring SNPs except for SNP5-SNP6 (*D*' = 0.286) in the control samples, suggesting that there may be a recombination hot spot present in the small region (7.8 kb) between the two SNPs (Table 3 - additional file 3. ). We plotted the magnitude of LD with the physical distance for each pair of the SNPs, and estimated the average half-length of LD to be 31.8 kb by assuming a linear regression (Fig. 2). This is approximately half of the previously estimated size 60 kb in a United States population of north-European descent [21].

Significant associations of schizophrenia with genotype (*P *= 0.013) and allele (*P *= 0.008) frequencies of SNP2 (rs4755404) were detected (Table 2 - additional file 2. ). However, none of these "single-marker" associations survived after Bonferroni corrections. An A-G transition in codon 206, causing a substitution of serine for asparagine, was identified in the exon 5 of *SLC1A2 *in a heterozygous sporadic ALS patient [29]. Since located in a putative glycosylation site, the nonsynonymous SNP is potentially involved in the pathophysiology of schizophrenia through affecting the glycosylation status and the transport activity of *SLC1A2 *[30]. No occurrence of the G allele of the SNP in 124 Italian schizophrenic and 50 control subjects has been reported [30]. We found also only A allele of the SNP in the 100 controls and 100 cases of the Kyushu samples (data not shown).

In pairwise haplotype association analyses, SNP2 consistently showed significant haplotype associations. The *P *value of the combination SNP2-SNP7 was still significant even after Bonferroni correction (*P *= 9.4 × 10^-5^, *P*_corr _= 0.0052). In our second sample set, the Aichi sample, no significant association of SNP2 was observed in any of the analyses of genotypes, alleles and haplotypes. Cumulative analyses of the two sample sets, however, provide the replication of the significant haplotype association of SNP2-SNP7 with schizophrenia (*P *= 5.0 × 10^-4^). The frequency of the G-C haplotype in schizophrenics (26.6%) was notably higher than in controls (5.6%), suggesting that the G-C haplotype may be a risk haplotype for schizophrenia. We observed that the G-C haplotype frequency of schizophrenics (20.0%) was only slightly higher than controls (14.2%) in the Aichi sample, suggesting a less contribution of this locus on schizophrenia pathogenesis in the Aichi sample, although no apparent difference in clinical subtypes between both sample sets studied in this paper. The positive association reported here needs to be validated in larger sample sets, and it would also be worthwhile to search for functional SNPs in the region spanning SNP2-SNP7.

## Conclusion

We concluded that at least one susceptibility locus for schizophrenia is probably located within or nearby *SLC1A2 *in the Japanese population.

## Competing interests

None declared.

## List of abbreviations used

SNP; single nucleotide polymorphism

DSM-IV; dianostic and statistical manual of mental disorders, 4^th ^edn

PCR; polymerase chain reaction

HWE; Hardy-Weinberg equilibrium

LD; linkage disequilibrium

EAAT; excitatory amino acid transporter

## Authors' contributions

XD carried out genotyping, statistical analyses and drafted the manuscript: HS participated in design of this study and statistical analyses: HN, NT, NI and NO participated in collecting specimens and clinical data: YF conceived of the study and participated in its design and coordination.

## Pre-publication history

The pre-publication history for this paper can be accessed here:



## Supplementary Material

Additional file 1 PCR primers for genotyping of SNPs in SLC1A2. Click here for file

Additional file 2 Genotype and allele frequencies of SNPs in SLC1A2 in Kyushu samples. Click here for file

Additional file 3 Pairwise linkage disequilibrium and haplotype association in SLC1A2. Click here for file

Additional file 4 Association analysis of the SNP2-SNP7 haplotype using two sample sets. Click here for file
